# Which Green Space Metric Best Predicts a Lowered Odds of Type 2 Diabetes?

**DOI:** 10.3390/ijerph18084088

**Published:** 2021-04-13

**Authors:** Soumya Mazumdar, Shanley Chong, Thomas Astell-Burt, Xiaoqi Feng, Geoffrey Morgan, Bin Jalaludin

**Affiliations:** 1 South Western Sydney Clinical School, University of New South Wales Medicine, Liverpool, NSW 2170, Australia; Shanley.Chong@health.nsw.gov.au; 2 Population Health Intelligence, South Western Sydney Local Health District, Liverpool, NSW 2170, Australia; Bin.Jalaludin@health.nsw.gov.au; 3 Population Wellbeing and Environment Research Lab (Power Lab), School of Health and Society, Faculty of Arts, Social Sciences and Humanities, University of Wollongong, Wollongong, NSW 2522, Australia; thomasab@uow.edu.au (T.A.-B.); xiaoqi.feng@unsw.edu.au (X.F.); 4 Menzies Centre for Health Policy, School of Public Health, University of Sydney, Sydney, NSW 2006, Australia; 5 Chinese Center for Disease Control and Prevention, National Institute of Environmental Health, Beijing 102206, China; 6 School of Public Health, Peking Union Medical College and The Chinese Academy of Medical Sciences, Beijing 102206, China; 7 School of Population Health, Faculty of Medicine and Health, University of New South Wales, Sydney, NSW 2052, Australia; 8 University Centre for Rural Health, School of Public Health, University of Sydney, Lismore, NSW 2480, Australia; geoffrey.morgan@sydney.edu.au

**Keywords:** green space, circular buffer, network buffer, health outcomes, type 2 diabetes

## Abstract

The choice of a green space metric may affect what relationship is found with health outcomes. In this research, we investigated the relationship between percent green space area, a novel metric developed by us (based on the average contiguous green space area a spatial buffer has contact with), in three different types of buffers and type 2 diabetes (T2D). We obtained information about diagnosed T2D and relevant covariates at the individual level from the large and representative 45 and Up Study. Average contiguous green space and the percentage of green space within 500 m, 1 km, and 2 km of circular buffer, line-based road network (LBRN) buffers, and polygon-based road network (PBRN) buffers around participants’ residences were used as proxies for geographic access to green space. Generalized estimating equation regression models were used to determine associations between access to green space and T2D status of individuals. It was found that 30%–40% green space within 500 m LBRN or PBRN buffers, and 2 km PBRN buffers, but not within circular buffers, significantly reduced the risk of T2D. The novel average green space area metric did not appear to be particularly effective at measuring reductions in T2D. This study complements an existing research body on optimal buffers for green space measurement.

## 1. Introduction

Residential environments have a significant influence on health outcomes [1,2,3]. The World Health Organization (WHO) reported that physical inactivity, linked to poor walkability and lack of access to recreational areas, accounts for 3.3% of global deaths [4]. In recent years, one potentially important residential environmental factor that has attracted global interest is green space [5,6]. Living in greener places is positively associated with a wide range of health indicators [7]. One of the potential mechanisms for these associations may be that green space prompts, facilitates, and reinforces location-specific physical activity [8]. Among the many health outcomes that green space related physical activity is associated to, of particular relevance, is the association with healthy blood glucose levels and reduction in the risk of type 2 diabetes (T2D) [9,10,11,12,13], with some cohort studies reporting a 10%–40% reduction in odds of T2D from accessible green space [13].

While green spaces may indicate a myriad of areas with vegetation ranging from nature strips and backyard gardens to public parks, in this paper we confine ourselves to green spaces that can be utilised to engage in meaningful physical activity or that can affect mental health. Thus, green spaces can be defined as outdoor sports fields, school playgrounds, any vegetated land adjoining an urban area including bushland, nature reserves, national parks, parks, and rural or semi-rural areas immediately adjoining an urban area [14].

To understand how access to green space impacts on health outcomes such as T2D, it is important to define a spatial unit that best represents an individual’s accessible neighbourhood green space. The choice of a spatial metric of access to green space may influence its relationship with health outcomes. For instance, in a landmark study, Oliver et al. found that using line-based road network buffers resulted in stronger associations between built environment attributes and walking than circular buffers [15]. Commonly used spatial metrics to measure access to green space are based on proximity measures, such as the “container approach” [15]. The container approach involves two key metrics. The first metric is an appropriate geographic container or spatial unit and the second is an appropriate green space metric within the container.

The choice of an appropriate geographic container or spatial unit is important as it can influence the exposure-outcome associations [15]. Some examples of spatial units are pre-defined small areas, circular buffers, and road network buffers. While pre-defined administrative areas such as shires, counties or local government areas are easily identifiable, replicable, and can be linked to a secondary data source such as the census [16], they may not replicate the actual green space experience of the person. This experience may be better represented by line based road network (LBRN) buffers, or by polygon-based road network (PBRN) buffers [15]. While the former assumes the road network around a person’s residence (and a small offset across the roads) to be part of a buffer, the later assumes that the area between the roads are also part of the buffer. Thus, for instance, while a typical jogger may utilise existing street networks for exercise making the LBRN buffer more relevant in his/her case, an off-road enthusiast may wish to run across fields and nature strips between roads making the PBRN buffer more relevant. The size of the buffer is also important, with, for instance, 500-meter (m) buffers representing the immediate area surrounding the residence, 1-kilometre (km) buffers representing a typical 10-min walk, and a 2-km buffer representing perhaps the maximum extent of walking from the home, or a reasonable distance for running and exercise [17,18].

Appropriately quantifying the amount of green space within a container is also important. Thus for instance, to quantify the amount of green space within a buffer, studies count the number of discrete green spaces within each buffer [19,20], while others calculate the actual surface area of the green spaces [21] or the proportion of green space within each buffer [22,23,24]. Since green spaces may come in various shapes and sizes, the latter (actual surface area or proportion of green space) is likely to provide a better estimate of green space than the former. However, even these measures suffer from a drawback. This drawback stems from the assumption that individuals stop walking or running at the boundary of the buffer. Thus, for instance, if half a percent of a buffer were to be covered by a park, but that park were to extend beyond the buffer for five square kms, complete with scenic jogging and walking paths, a possible incorrect assumption would be made that an individual residing in the buffer has limited green space access, with the attractiveness of the additional green space beyond the buffer boundary encouraging physical activity beyond the boundary. To examine this issue, we developed a novel metric of green space access. This metric, which we call “average contiguous green space area” or ACGSA, is based on the portion of green space within a given buffer and any contiguous portion of that particular green space that may lie outside the buffer.

The goal of this study thus, is to explore the consistency in direction and magnitude of associations using different buffer types (circular, LBRN, PBRN), buffer sizes (500 m, 1 km, 2 km), and green space metrics (percent green space or ACGSA) as a proxy of access to green space [25]. A secondary goal is to discover and discuss the metric that produces the strongest association between these green space measures and the prevalence of T2D. This is realized using data from a large cohort study which is described in detail in the next section.

## 2. Method

### 2.1. Study Population

The study area was the Sydney Statistical Division (SSD), in New South Wales (NSW), Australia, which had a population of approximately 4.42 million in 2016 and covers an area of 12,137 square kms [26]. We obtained T2D and relevant covariate data at the individual level from the Sax Institute’s 45 and Up Study. The 45 and Up Study is a population-based cohort survey of NSW residents aged 45 years and older undertaken between 2006 and 2009. Potential participants were randomly selected from the Medicare Australia database (Australia’s universal publicly funded health insurance scheme). The response rate was 18% and participants comprised 11% of the NSW population aged 45 years and older [27]. Participants joined the study by completing a mailed self-administered questionnaire and providing consent for long term follow-up, including linkage to personal health records. The full study cohort consisted of 267,153 people aged 45 years or older at the time of recruitment, subsetting which to the Sydney SSD resulted in 94,075 records.

The 45 and Up Study was approved by the University of New South Wales Human Research Ethics Committee and the University of Sydney Human Research Ethics Committee.

### 2.2. Access to Green Space

Green space data were obtained from Pitney Bowes Australia StreetPro 2016. As mentioned earlier, in this dataset, green space includes national parks, nature reserves, historic sites, state forests, state recreation areas, wildlife refuges, conservation parks, protected areas, wildlife reserves, urban recreation parks and other urban green spaces.

The proportion of green space within 500 m, 1 km, and 2 km circular buffers, LBRN buffers and PBRN buffers around participants’ residences were used as proxies for geographic access to green space. Circular buffers of the specified radii were created around the centroid of each participant’s residence location. LBRN buffers were created using the Pitney Bowes StreetPro 2016 data, which also included all roads in the study area excluding highways. We used ArcGIS network analyst [28] to calculate the specified line-based buffer minus 50 m, resulting in 450 m, 950 m and 1.95 km buffers along the road network from each participant’s residence. A 50 m simple buffer was then constructed around this line-based buffer, resulting in 500 m, 1 km, and 2 km buffers around each participant’s residence constrained to the contiguous road network. A previous study of the influence of land use on walking suggested that the use of a 50 m buffer along the road network is sufficient to capture green space along the selected roads [15].

The PBRN buffers were also created using the StreetPro road network data and ArcGIS network analyst [28]. We calculated the endpoints of all possible routes up to the specified distance (500 m, 1 km, and 2 km) along the road network for each participant’s residence. The endpoints were then connected to form irregular polygons.

Two proxy measures of green space were then created using the specified buffers: proportion of green space and ACGSA. The proportion of green space within the specified buffers were categorised into 0%–10%, 10%–20%, 20%–30%, 30%–40%, and >40%. Note that we use standard mathematical interval notation, where for instance, 10%–20% implies that the category ranges from 10% (inclusive) to less than 20%.

To evaluate ACGSA we first calculated the number of green space parcels that overlapped the specified buffers. Green space polygons which were completely or partially situated inside the buffers were included. The total area of these green space parcels including those partially located outside the buffers were calculated. The average area within these green space polygons was then calculated.

**Figure 1:**
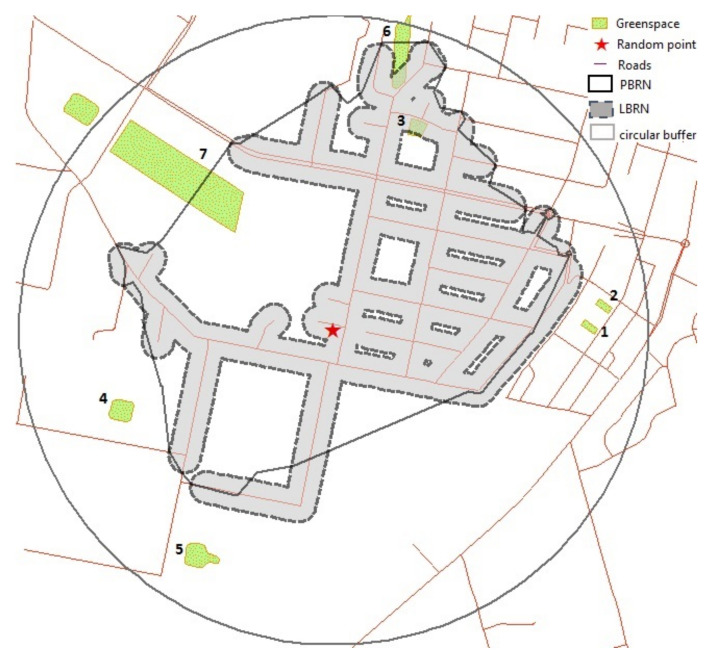
Comparison of different buffers used in green space metrics.

An example is provided in Figure 1 which displays seven green space polygons located within the 1 km circular buffer, of which three green space polygons are located within a PBRN buffer and two green space polygons are located within a LBRN buffer around a random point. The total area of the smallest green space polygon (green space 1) is 985 m^2^ followed by 1000 m^2^ (green space 2), 2364 m^2^ (green space 3), 4267 m^2^ (green space 4), 5542 m^2^ (green space 5), 9011 m^2^ (green space 6) and the largest green space polygon (green space 7) is 54,275 m^2^. The ACGSA within the 1 km circular buffer is thus 0.011 km^2^, 0.022 km^2^ for the 1 km PBRN buffer, and 0.006 km^2^ for 1 km LBRN buffer.

Average green space area was grouped into distinct categories. Since the percentage of average green space area over 50 km^2^ ranged from 1.1% to 1.8% for 500 m buffers, 1.6% to 2.6% for 1 km buffers, and 2.9% to 4.2% for 2 km buffers, we categorized this variable into the following categories:

(0, 0.5 km^2^], (0.5, 1 km^2^], (1, 3 km^2^], (3, 5 km^2^], (5, 10 km^2^], (10, 20 km^2^], (20, 30 km^2^], (30, 40 km^2^], and (40, 50 km^2^].

### 2.3. Outcome Variables

Prevalence of T2D was defined using participants who self-reported T2D in the 45 and Up Study. The relevant questions to determine a diagnosis of T2D in the survey were “Has a doctor ever told you that you have diabetes?” and “Have you taken Diabex, Diaformin, Metformin for most of the last 4 weeks?”. Self-reported diagnosis of T2D in the 45 and Up Study has high sensitivity (83.7%) and specificity (97.7%) compared to administrative hospitalisation data [29].

### 2.4. Covariates

Participant reported socio-demographic characteristics, including age, gender, body mass index (BMI), marital status, educational attainment (university/Technical and Further Education (TAFE), high school and less than 10 years of schooling), ancestry (English speaking countries, Europe, Middle East, Asia, and Other) and employment status were included as covariates in the regression models. We also included physical functioning (measured using the Medical Outcomes Study (MOS) Physical Functioning Scale; it ranges from 0 to 100 and was categorised into no limitation (100), minor limitation (95–99), moderate limitation (85–94), or severe limitation (0–84)) [30], psychological distress (Kessler-10 (K10); a K10 score of ≥22 reflects high or very high psychological distress) [31] and an area-level deprivation score as covariates in the model.

Area-level deprivation was measured by the 2006 Index of Relative Socio-Economic Disadvantage (IRSED) quintiles at the postcode level. The IRSED was created by the Australian Bureau of Statistics to compare social and economic disadvantage across geographical areas in Australia. The index is derived from the 2006 Census variables such as low income and educational attainment, high unemployment, and people working in unskilled occupations [32,33].

Moderate to vigorous physical activity (MVPA; minutes per week) and sitting (hours per week) were also included in the model as covariates. Physical activity was assessed in the 45 and Up study using questions from the Active Australia Survey [34] which have acceptable reliability [35] and validity [36]. In this instrument, walking is defined as walking for recreation or exercise or to get to or from places. Vigorous physical activity refers to any activity that causes a participant to breathe harder or puff and pant. Moderate physical activity refers to gentle exercise like gentle swimming, social tennis, vigorous gardening or work around the house. Total minutes of MVPA per week is calculated by the sum of minutes walking, moderate physical activity and twice the minutes of vigorous physical activity [34].

### 2.5. Statistical Analysis

We used generalised additive models (GAMs) to explore the relationships between the green space metrics and the prevalence of T2D, and to categorise green space metrics based on points of inflexion in the nonlinear relationships (not shown). We expect individuals in the same geography (local government area) to behave similarly. Thus, we used generalized estimating equations (GEE) which include an additional variance component to accommodate for correlated data and to allow for differences among local government areas. GEE logistic regression models were used to determine associations between access to green space and T2D prevalence. We assumed a compound-symmetric (exchangeable) correlation structure for the GEE model. Since GEE is a non-likelihood-based method, QIC (quasi-likelihood under independence model criterion) was used for variable selection and selecting the working correlation matrix. A lower QIC value indicates better model fit. As T2D may lead to physical disability, participants with severe physical functional limitation were excluded when fitting the GEE models.

## 3. Results

Of the 94,075 participants, 7192 (7.65%) participants reported T2D. Participants who were male, older, reported non-English ancestry, had lower education qualification, lived in more disadvantaged areas, were unemployed, obese, reported psychological distress, were engaged in less amount of time in MVPA, and spent more time in sitting were more likely to report T2D (Table 1).

Descriptive statistics for the various green space metrics are presented in the Table S1. For each buffer size, the majority of PBRN and LBRN buffers contain a smaller percentage of green space and ACGSA than circular buffers.

**Figure 2:**
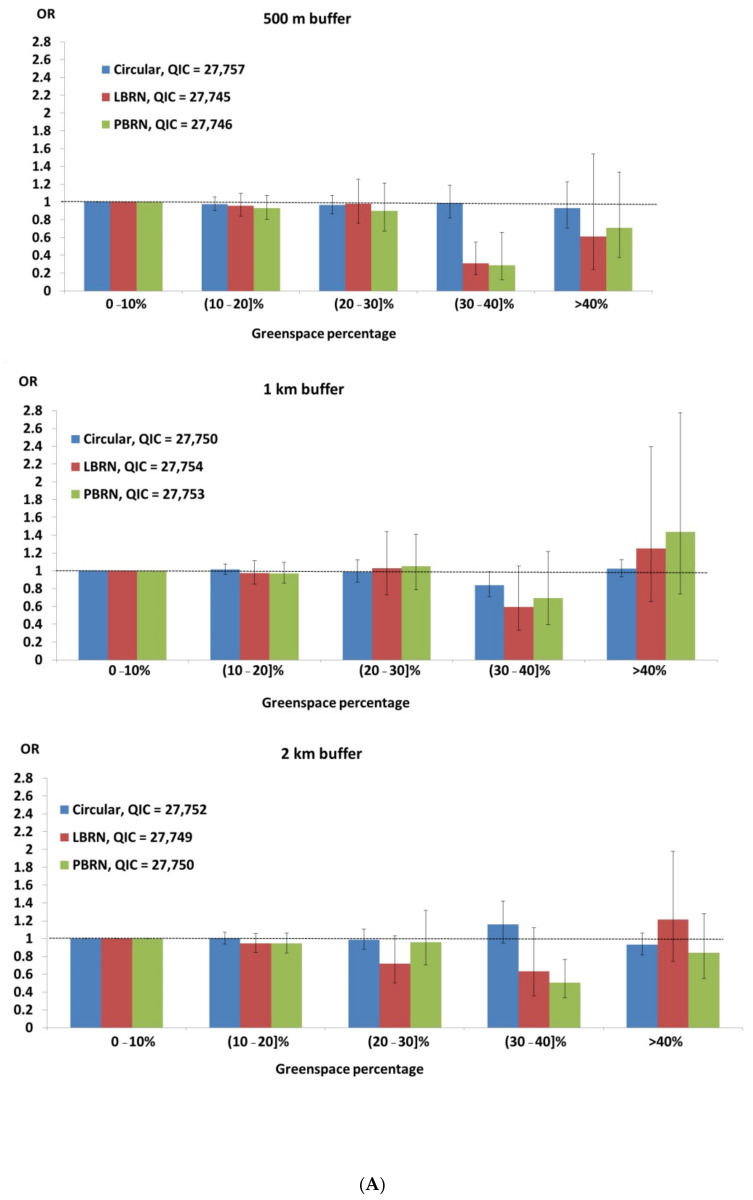

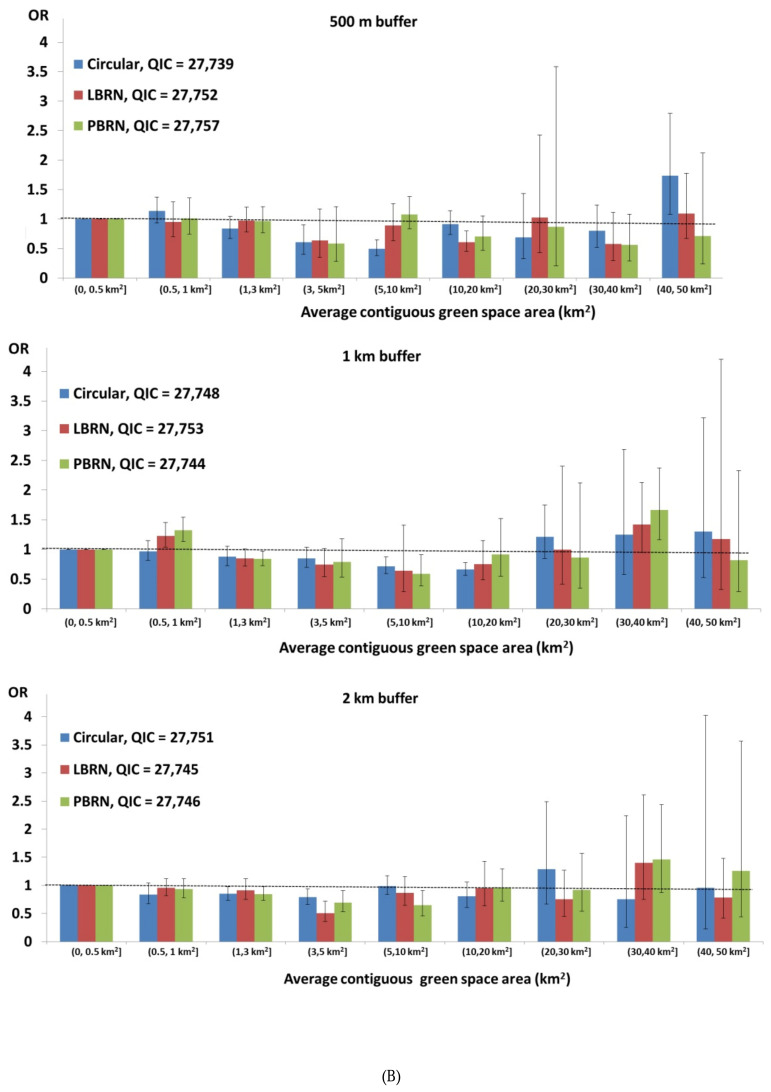
(**A**): Odds of T2D by different green space metrics (green space percentage). Abbreviations-LBRN: Line based network buffer, PBRN: Polygon-based road network buffer, OR: Odds Ratio and QIC: Quasi-likelihood under Independence model Criterion. (**B**): Odds of T2D by different green space metrics ((Average contiguous green space area). Abbreviations-LBRN: Line based network buffer, PBRN: Polygon-based road network buffer, OR: Odds Ratio and QIC: Quasi-likelihood under Independence model Criterion.

Results from GEE models for the risk of T2D by the percentage of green space within the various buffer types and sizes are presented in Figure 2A (green space percentage) and Figure 2B (ACGSA). The percentage of green space ≤10% and average green space area of ≤0.5 km^2^ were used as reference groups. There were significant reductions in odds of T2D when 30%–40% of LBRN or PBRM buffers of 500 m radius, or PBRN buffers of 2 km radius were covered with green space. A U-shaped pattern in odds ratios is also apparent, across all buffer sizes, with a dip in the odds ratios at 30%–40% and peaks on either side at 20%–30% and greater than 40%. The models with the 500 m and 2 km LBRN buffers also had the lowest QIC in their respective buffer size groups.

Circular buffers with 5–10 km^2^ of ACGSA were associated with a significant reduction in T2D odds when buffer sizes are less than 2 km. The models with ACGSA in circular buffers less than 2 km in diameter also have the lowest (500 m buffer) and second lowest (1 km buffer) QICs. A general U-shaped trend was also visible with the odds of diabetes dipping when the average green space area in the buffer ranged from 3–20 km^2^.

## 4. Discussion

Our paper is the first to investigate the relationship between three different buffer types and two different ways of measuring green space on T2D, including a novel metric of average contiguous green space area. We report three significant findings. First, we showed that while using percent green space in LBRN and PRBRN buffers for analyses showed similar reduction in diabetes odds, circular buffers tended to show lower reduction in odds compared to LBRN and PBRN buffers. Second, the optimal amount of green space in buffers appears to be between 30% and 40% with increased odds of diabetes with either more or less green space. Third, the optimal amount of average contiguous green space area for reducing the odds of T2D was found to be between 3 km^2^ to 20 km^2^ with 5–10 km^2^ of average green space within circular buffers showing the greatest reduction in odds.

This study has a number of strengths. First, it introduces novel comparisons and an entirely new metric. Second, it leverages a large survey dataset from a diverse metropolitan area. Finally, it accounts for a number of lifestyle factors and demographic factors at individual level. We adjusted for socio economic status which has been previously found to confound the relationship between diabetes [37] and green space [38]. However, our study also suffers from several limitations. The health data and land-use data are from different time periods, and some differences in actual exposures may be present. However, since green spaces often change over long secular time periods, it is unlikely that this will affect the overall findings of our study. The green space included state forests and national parks which may not be readily accessible to the public. A more useful categorization of green space would have been into more usable categories, such as, sports fields, bushland, presence of picnic facilities, etc. A further limitation is that we did not have information about the quality and attributes of the green space. Smaller public open space with more attributes attract more users and presence of trees, water (e.g., a lake), park maintenance, and the availability of amenities such as outdoor fitness equipment are also important factors influencing use of green space [39]. It is the attributes of green space that determine the use of green space for promoting physical activity and better mental health. In addition, we have not adjusted for other neighbourhood features such as walking paths along rivers and beaches which may promote physical activity. Another limitation is that, residential history was not reported in the survey and we are unable to account for reverse causality. A final limitation is that some of the odds ratios, especially for the larger buffer sizes, had sizeable uncertainties associated with them (large confidence intervals), resulting from sparse data. Thus, these results should be interpreted with caution.

Thirty to forty percent green space within 500 m LBRN or PBRN buffers, and 2 km PBRN buffers, but not within circular buffers significantly reduces the risk of diabetes. This underscores the importance of accessible green space within close proximity to residence in reducing the odds of diabetes, and the importance of LBRN and PBRN in measuring this. This is consistent with a study by Maas et al., [40] which found that prevalence of T2D is significantly lower in living environments with more green space within a 1 km radius. Similarly, another study reports significantly lowered incidence of psychological distress when green space in the neighbourhood exceeds 30% [41]. The presence of 40% or more green space in a city block has been associated with the greatest reduction in temperatures, which may facilitate green exercise, and also support mental health [42]. A recent systematic review also substantiates the above findings [13]. Finally, our research complements findings from another recent review that green space within 2 kms from home are appropriate for exploring relationships with physical health [43].

Circular buffers capture a larger area than LBRN or PRBN buffers, and may be able to capture multiple entrance points to extended regions of green space such as national parks located outside the buffer area, and thus be more relevant to the average green space area metric [44]. We found significantly lower diabetes odds with 5–10 km^2^ of average green space within circular buffers. These are very large green space areas, and since corresponding LBRN and PBRN buffers do not show a relationship, this may imply that these areas are not necessarily accessible to the resident. Thus, the reduced odds of diabetes are likely through the mental health, reduced stress and better sleep related pathways, that are manifested from living close to large green space areas [45]. It does appear, however, that the average contiguous green space area metric is not appropriate for assessing reduction in T2D, with standard network buffers performing better than this metric. Also, the LBRN and PBRN buffers appear to be performing equally well. PBRN buffers are better suited for densely settled areas with high street connectivity, while LBRN buffers perform better in sparsely settled areas with low street connectivity [15]. Most of metropolitan Sydney is densely settled, and thus, in a global analysis such as this, the comparative advantage of using LBRN buffers is lost with both buffers yielding similar results.

A U-shaped trend was visible for analyses using both average contiguous green space area and with percent green space. This is expected, given that small patches of green space may be of limited utility, and very large green spaces may be perceived to be unsafe. This may be mediated through the relative paucity of park features associated with safety, such as good lighting, visibility of houses, and high trafficked roads, all of which are rarely found in very large green spaces such as national parks and large undeveloped areas with vegetation [46].

## 5. Conclusions

While, as expected, the associations between T2D and green space varied across the different buffer types, this study indicates that network buffers are optimal in analyses of T2D and green space relationships. Second, this study found that 30%–40% green space within walkable distance from home is optimal for the reduction of T2D odds. Third, large contiguous green spaces that extend beyond buffer boundaries may have limited influence on T2D odds. Future studies on the effect of different buffers in green space and health evaluation could focus on newer green space metrics such as google street view that provide a more realistic estimate of the individual’s green space experience.

## Figures and Tables

**Table 1:** Descriptive statistics of the study population (*n* = 94,075).

	*n*	Prevalence of T2D; *n* (%)	OR	95% CI
**Sex**				
Female	49,256	2916 (5.9)	0.57	0.54–0.60 ^1^
Male	44,819	4276 (9.5)	1	
**Age-group (years)**				
≤55	29,120	1157 (3.9)	1	
>55–65	29,406	2178 (7.4)	1.94	1.80–2.09 ^1^
>65–75	17,547	1925 (11.0)	2.93	2.61–3.30 ^1^
>75	18,002	1932 (10.7)	2.94	2.59–3.33 ^1^
**Living with partner**				
Yes	24,628	2127 (8.6)	0.81	0.76–0.87 ^1^
No	69,447	5065 (7.3)	1	
**Ancestry**				
Middle East	712	91 (12.8)	1.89	1.32–2.70 ^1^
Asia	5095	571 (11.2)	1.73	1.50–2.01 ^1^
Other	13,602	1162 (8.5)	1.28	1.15–1.42 ^1^
Europe	11,427	903 (7.9)	1.11	0.99–1.25 ^3^
English speaking countries	63,239	4465 (7.1)	1	
**Educational qualification** (missing = 1401)				
University/TAFE	46,544	2871 (6.1)	0.63	0.57–0.69 ^1^
High school	19,150	1634 (8.5)	0.88	0.82–0.95 ^1^
Less than 10 years of schooling	26,980	2561 (9.5)	1	
**IRSED quintiles** (missing = 16)				
(Most disadvantaged) 1st quintile	8979	1160 (12.9)	1	
2nd quintile	9516	914 (9.6)	0.72	0.64–0.82 ^1^
3rd quintile	10,497	1016 (9.8)	0.72	0.64–0.80 ^1^
4th quintile	24,468	1837 (7.5)	0.55	0.49–0.62 ^1^
(Least disadvantaged) 5th quintile	40,599	2265 (5.6)	0.39	0.34–0.45 ^1^
**Unemployed**				
No	91,714	6962 (7.6)	0.74	0.64–0.86 ^1^
Yes	2361	230 (9.7)	1	
**BMI** (missing = 5630)				
Under weight	1309	41 (3.1)	0.21	0.15–0.29 ^1^
Normal weight	34,700	1500 (4.3)	0.25	0.23–0.27 ^1^
Over weight	34,049	2469 (7.3)	0.44	0.41–0.46 ^1^
Obese	18,387	2755 (15.0)	1	
**Psychological distress** (missing = 2353)				
No	84,770	6161 (7.3)	0.57	0.52–0.62 ^1^
Yes	6952	815 (11.7)	1	
**Physical functional limitation** (missing = 8771)				
None	29,845	1178 (4.0)	0.17	0.16–0.19 ^1^
Minor	29,378	1962 (6.7)	0.30	0.28–0.33 ^1^
Moderate	19,261	2226 (11.6)	0.55	0.50–0.61 ^1^
Severe	6820	1248 (18.3)	1	
**Less than 5 daily portions of fruit and vegetables**				
No	56,542	4252 (7.5)	0.96	0.89–1.02 ^3^
Yes	37,533	2940 (7.8)	1	
**MVPA deciles** (missing = 10,422)				
1st (least MVPA)	8168	1040 (12.7)	2.45	2.17–2.77 ^1^
2	9136	912 (10.0)	1.83	1.62–2.07 ^1^
3	7451	611 (8.2)	1.50	1.33–1.68 ^1^
4	8412	635 (7.6)	1.32	1.15–1.52 ^1^
5	8459	573 (6.8)	1.19	1.05–1.36 ^2^
6	8657	548 (6.3)	1.11	0.99–1.24 ^3^
7	8312	509 (6.1)	1.07	0.93–1.24 ^3^
8	8932	547 (6.1)	1.10	0.99–1.23 ^3^
9	7785	449 (5.8)	0.99	0.86–1.14 ^3^
10th (most MVPA)	8341	482 (5.8)	1	
**Sitting time deciles**(missing = 7026)				
1st (Least time sitting)	11,505	791 (6.9)	0.82	0.70–0.97 ^2^
2	9077	622 (6.9)	0.84	0.71–0.99 ^2^
3	117	7 (6.0)	0.72	0.35–0.68 ^3^
4	13,811	1072 (7.8)	0.92	0.81–1.03 ^3^
5	9413	696 (7.4)	0.88	0.77–1.01 ^3^
6	12,458	978 (7.9)	0.93	0.82–1.06 ^3^
7	4848	325 (6.7)	0.78	0.64–0.96 ^2^
8	9653	717 (7.4)	0.88	0.77–1.01 ^3^
9	9059	724 (8.0)	0.97	0.85–1.10 ^3^
10th (Most time sitting)	7108	595 (8.4)	1	

^1^*p*-value < 0.001, ^2^ *p*-value < 0.05, ^3^ *p*-value > 0.05. BMI = Body Mass Index; MVPA = Moderate to Vigorous Physical Activity; IRSED = Index of Relative Socio-Economic Disadvantage, OR = Odds Ratio, CI = Confidence Interval, T2D = Type 2 Diabetes, TAFE = Technical and Further Education.

## Supplementary Materials

The following are available online at https://www.mdpi.com/article/10.3390/ijerph18084088/s1, Table S1: Characteristics of green space by green space metrics, *n* = 94,075.
