# Supplementary material for: Which Green Space Metric Best Predicts a Lowered Odds of Type 2 Diabetes?
Source: Int J Environ Res Public Health. 2021 Apr 13;18(8):4088. doi: 10.3390/ijerph18084088 (PMC8068830; doi:10.3390/ijerph18084088)
Supplement: Supplementary file 1 [file ijerph-18-04088-s001.pdf]

Table S1. Characteristics of green space by green space metrics, n = 94,075.

Green Space %	Type of Buffer, *n* (%)		
**Within 500m buffer**	**Circular**	**PBRN**	**LBRN**
(0–10%]	59,549 (63.3)	85,537 (90.9)	82,489 (87.7)
(10–20%]	20,885 (22.2)	7033 (7.5)	9561 (10.2)
(20–30%]	7902 (8.4)	1068 (1.1)	1411 (1.5)
(30–40%]	3669 (3.9)	241 (0.3)	368 (0.4)
>40%	2070 (2.2)	196 (0.2)	246 (0.3)
**Within 1km buffer**			
(0–10%]	51,025 (54.2)	82,977 (88.2)	82,415 (87.6)
(10–20%]	27,262 (29.0)	9803 (10.4)	10,2561 (10.9)
(20–30%]	9762 (10.4)	846 (0.9)	891 (0.9)
(30–40%]	3458 (3.7)	288 (0.3)	304 (0.3)
>40%	2568 (2.7)	161 (0.2)	209 (0.2)
**Within 2km buffer**			
(0–10%]	41,307 (43.9)	78,368 (83.3)	81,833 (87.0)
(10–20%]	37,907 (40.3)	13,859 (14.7)	10,739 (11.4)
(20–30%]	9090 (9.7)	1285 (1.4)	954 (1.0)
(30–40%]	3141 (3.3)	324 (0.3)	311 (0.3)
>40%	2630 (2.8)	239 (0.3)	238 (0.3)
**Average green space area**	**Type of buffer, n (%)**		
**Within 500m**	**Circular**	**PBRN**	**LBRN**
**≤0.5km²**	87,070 (92.6)	88,901 (94.5)	88,842 (94.4)
**(0.5–1] km²**	2628 (2.8)	2188 (2.3)	2248 (2.4)
**(1–3] km²**	1959 (2.1)	1425 (1.5)	1404 (1.5)
**(3–5] km²**	551 (0.6)	413 (0.4)	415 (0.4)
**(5–10] km²**	631 (0.7)	344 (0.4)	326 (0.3)
**(10–20] km²**	563 (0.6)	454 (0.5)	489 (0.5)
**(20–30] km²**	244 (0.3)	98 (0.1)	111 (0.1)
**(30–40] km²**	289 (0.3)	172 (0.2)	161 (0.2)
**(40–50] km²**	140 (0.1)	80 (0.1)	79 (0.1)
**Within 1km**			
**≤0.5km²**	86,156 (91.6)	87,913 (93.4)	87,965 (93.5)
**(0.5–1] km²**	2520 (2.7)	2317 (2.5)	2254 (2.4)
**(1–3] km²**	1959 (2.1)	1710 (1.8)	1702 (1.8)
**(3–5] km²**	1089 (1.2)	480 (0.5)	488 (0.5)
**(5–10] km²**	976 (1)	602 (0.6)	510 (0.5)
**(10–20] km²**	650 (0.7)	490 (0.5)	607 (0.6)
**(20–30] km²**	403 (0.4)	293 (0.3)	301 (0.3)
**(30–40] km²**	235 (0.2)	190 (0.2)	166 (0.2)
**(40–50] km²**	87 (0.1)	80 (0.1)	82 (0.1)
**Within 2km**			
**≤0.5km²**	84,290 (89.6)	87,133 (92.6)	87,134 (92.6)
**(0.5–1] km²**	2070 (2.2)	1943 (2.1)	1788 (1.9)
**(1–3] km²**	3619 (3.8)	2042 (2.2)	2165 (2.3)
**(3–5] km²**	1471 (1.6)	824 (0.9)	919 (1)
**(5–10] km²**	1420 (1.5)	806 (0.9)	704 (0.7)
**(10–20] km²**	806 (0.9)	802 (0.9)	895 (1.0)
**(20–30] km²**	148 (0.2)	244 (0.3)	173 (0.2)
**(30–40] km²**	159 (0.2)	195 (0.2)	178 (0.2)
**(40–50] km²**	92 (0.1)	86 (0.1)	119 (0.1)
